# Ear Recognition from One Sample Per Person

**DOI:** 10.1371/journal.pone.0129505

**Published:** 2015-05-29

**Authors:** Long Chen, Zhichun Mu, Baoqing Zhang, Yi Zhang

**Affiliations:** 1 School of Automation and Electrical Engineering, University of Science and Technology Beijing, Beijing, China; 2 No.208 Institute, the Second Academy of CASIC, Beijing, China; University of Chinese Academy of Sciences, CHINA

## Abstract

Biometrics has the advantages of efficiency and convenience in identity authentication. As one of the most promising biometric-based methods, ear recognition has received broad attention and research. Previous studies have achieved remarkable performance with multiple samples per person (MSPP) in the gallery. However, most conventional methods are insufficient when there is only one sample per person (OSPP) available in the gallery. To solve the OSPP problem by maximizing the use of a single sample, this paper proposes a hybrid multi-keypoint descriptor sparse representation-based classification (MKD-SRC) ear recognition approach based on 2D and 3D information. Because most 3D sensors capture 3D data accessorizing the corresponding 2D data, it is sensible to use both types of information. First, the ear region is extracted from the profile. Second, keypoints are detected and described for both the 2D texture image and 3D range image. Then, the hybrid MKD-SRC algorithm is used to complete the recognition with only OSPP in the gallery. Experimental results on a benchmark dataset have demonstrated the feasibility and effectiveness of the proposed method in resolving the OSPP problem. A Rank-one recognition rate of 96.4% is achieved for a gallery of 415 subjects, and the time involved in the computation is satisfactory compared to conventional methods.

## Introduction

With recent developments in human society, traditional authentication methods, such as card- or password-based methods, can no longer fulfill the need for reliable and convenient human identification. Biometrics, which utilizes the physical or behavioral characteristics of human beings [1], provides a relatively new perspective. Whereas traditional authentication systems require users to carry ID cards and passwords that are easily forgotten, in biometrics, authentication is intrinsic to the user, and there is no concern regarding the inconvenience caused by poor memory. Although there are many biometric traits (e.g., fingerprint, face, iris), the ear has certain advantages over other traits [2].

The ear, which has been observed to vary significantly among individuals [3], has been proved to be a reliable method of human identification [4]. It is widely accepted that the human ear is invariant to facial expressions and aging [5] in comparison to the face. In addition, its relatively small size makes it inexpensive for storage and computation.

In real-world applications, several problems appear in biometric-based recognition systems. One such issue is the OSPP problem, i.e., only one training sample per person is available in the gallery. For applications such as identifying terrorists, law enforcement, driver’s license or passport card identification, it is often an unfortunate fact that few samples per person are available in the gallery for the task of recognition. In some cases, there is only one sample stored in the gallery, which is a significant challenge for an authentication system. Under this specific condition, particularly in large-scale identification applications, most of the traditional methods would suffer a serious decrease in performance or even fail to work. [6] The OSPP problem is not only an extreme example of the small-sample problem but also a more severe problem that must be carefully considered. Although the OSPP scenario is often avoided, it does have its benefits. As the training sample number decreases to its minimum (only one), the storage requirements and computational overhead also decrease. In a sense, this is advantageous for a recognition system, particularly for systems that deal with a large volume of registered persons. If there are no other choices, the OSPP problem must be resolved, and solving it would be of significant help in the biometric identification field.

Because 3D data contain more information and are less sensitive to illumination and occlusion than 2D images, there is a trend to use 3D data to improve recognition performance. With the development and popularization of 3D technology, the market has witnessed a decline in the cost of 3D sensors, making them an affordable solution for human authentication systems. Ear matching based on 3D data could achieve a higher accuracy than matching using only the corresponding 2D images [7]. A 2D texture image is typically acquired along with a 3D range image at the same time using a 3D scanner (e.g., Minolta VIVID series scanners). It would be a considerable loss of information for identification if we were to use the 3D data only. Because the OSPP problem is rooted in the lack of available information, fusing 2D and 3D information is an effective method of solving it.

In this paper, we propose a multi-keypoint descriptor sparse representation-based classification (MKD-SRC) based ear recognition scheme that requires only OSPP in the gallery. Instead of using only 2D data or only 3D data, we utilize a hybrid of 2D and 3D data to achieve better performance. We call this proposed method the hybrid MKD-SRC. To the best of our knowledge, this is the first study to consider SRC-based ear recognition with only OSPP enrolled in the gallery. Furthermore, this is also the first study to fuse 2D and 3D information into the SRC framework for ear recognition. The remainder of this paper is organized as follows. Section 2 presents the related studies of ear recognition. In Section 3, the hybrid MKD-SRC approach is proposed for ear recognition with only OSPP in the gallery. Experimental results and further discussion are presented in Section 4. Finally, we conclude this paper and discuss the direction of future studies in Section 5.

## Literature Review

Psychological experiments show that the human vision system is sensitive to changes in curvature and can extract salient points based on surface curvature to break complex shapes down into individual parts to perform the identification [8]. The human ear can be viewed as an approximate rigid biometrics whose structure contains a wealth of curvature information that can be used for identification. Many previous studies have been proposed for ear recognition, and some of them reported good performance. However, as discussed herein, the OSPP situation is common in real-world applications. Unfortunately, most of the traditional ear recognition systems require multiple samples for training or registration in the gallery.

Moreno *et al*. [9] developed the first fully automated system for ear recognition. Multiple features and several neural classifiers were used for classification. They developed an ear database of 168 images pertaining to 28 subjects (i.e., 6 samples per person). With 3 images per subject used for training, a recognition rate of 93% was achieved for the best of the three approaches. Mu *et al*. [10] extended this method by combining the outer ear shape with the inner ear structure. They also constructed an ear database composed of 308 images of 77 subjects. The training set was composed of OSPPs, and the recognition rate was 85%. As seen from the comparison of these two results, the recognition rate decreases sharply when the number of training samples decreases from 3 to 1. Yuizono *et al*. [11] proposed an ear recognition system that applied a genetic algorithm (GA). It evaluated the identity of the probe sample by measuring the mean square error between the probe sample and gallery sample. They obtained a rank-one recognition rate of 100% on a database containing 110 persons and six samples for each person.

Later, some successful approaches used in the area of face recognition were implemented in ear recognition. Principal component analysis (PCA) and linear discriminant analysis (LDA) are two well-known examples. Turk and Pentland [12] proposed the famous Eigenfaces method for face recognition in 1991. Then, a similar “eigen-ear” [13, 14] method was explored for ear biometrics. Yang *et al*. proposed 2D PCA, which improved the original PCA and gained attention due to its robustness to pose and illumination changes. Some efforts were made for LDA in ear recognition as well [15–17]. Although these studies reported good performance, they all required multiple samples for training.

When only one sample is available, local information becomes a useful tool. Scale invariant feature transform (SIFT), a well-known local descriptor, has been proven effective and robust in object recognition. The SIFT descriptor has been introduced in different biometric traits [18–22]. For ear recognition, Dewi and Yahagi [22] extracted keypoints by performing SIFT, and approximately 16 keypoints were generated for each sample. By simply calculating the number of matched keypoints and the average distance, they achieved a recognition rate of 78.8% with only one training sample per person. Later, Kisku *et al*. [21] used the SIFT descriptor to represent the structure of an ear sample. Ear regions were segmented manually, and the SIFT keypoints were detected only from the color slice regions. By combining SIFT features and a color homogeneity model, they reported a higher accuracy of 96.93% using the nearest neighbor approach.

In the milestone masterpiece of Wright *et al*. [23], a general classification algorithm SRC (sparse representation-based classification) for facial recognition was proposed, which provided new insights into biometric-based recognition. The SRC algorithm takes a probe image as a linear representation of all atoms in the dictionary, which is equal to the gallery. Theoretically, the coefficients of the linear representation should be sparse, with the nonzero coefficients on the same subject as the probe. Then, the identity of the probe is determined by minimizing the reconstruction error. Inspired by this study, a number of variants were proposed for different biometric traits. Naseem *et al*. [24] applied SRC to ear recognition with three to four samples per person in the gallery. Further, Kumar and Chan [25] proposed to exploit SRC for finite random transform-based local orientation information. Zhang *et al*. [26] used the scale information of Gabor wavelets in SRC-based ear recognition. Non-negative constraints on the dictionary and sparse coding coefficients were added to avoid canceling each other out by subtraction. Experimental results showed that this algorithm exhibited great robustness against occlusion. Zhang *et al*. [27] extended SRC to the field of 3D ear identification. 3D information was used as range images in their study, and the best recognition rate was 95.23%. Although these SRC-based methods achieved good performance, they have one common feature: they all require at least 5 samples per person [27] in the gallery to form an over-complete dictionary. Without a sufficient number of samples, the recognition rate declines sharply.

When the OSPP problem began to draw attention from researchers, it was 3D information that was the focus of many solutions. 3D data have an advantage over the 2D data in addressing lighting variation and pose changes [28]. From a different perspective, 3D data can provide more information than 2D data. Chen and Bhanu [29] proposed a complete ear recognition system that represented 3D ears using local surface patch (LSP). A modified iterative closest point (ICP) algorithm [30] was adopted to match the probe sample with all samples in the gallery. In their later study [31], a new dimension reduction algorithm was adopted, and the similarity between a model-test pair was computed in a low-dimensional space. They reported a recognition rate of 96.7% in the best case. Yan and Bowyer [32] later proposed an automatic ear recognition method using both 2D and 3D information. After the ear region was segmented from a profile image, an ICP-based approach was also used for 3D shape matching. This method achieved a rank-one recognition rate of 97.8%. Islam *et al*. [33] proposed a fast 3D local feature matching and fine matching method via ICP. It is superior to Yan and Bowyer’s method in terms of computational speed. These ICP-based methods required only one sample (or model) per person in the gallery, but the computation involved in the ICP algorithm is considerably intensive, and the ICP algorithm easily falls into local minima. In addition, the 3D point cloud data require considerable storage. For instance, a segmented ear point cloud data (as in form of. txt) is 536 kB, whereas its corresponding range image (as in form of. png) is only 10.1 kB. Thus, under the premise of satisfying performance, the range image is a better choice for 3D data representation.

Liao *et al*. [34] proposed a brand new method to address the OSPP problem in face recognition. They improved the SRC method by detecting keypoints in OSPP to form a multi-keypoint dictionary. In this manner, each subject in the gallery is represented by a group of descriptors (the number of each subject can vary), which made it possible to adjust the SRC method to the OSPP problem. Additionally, this method does not require an alignment, and partial data and occlusion are not an issue because the descriptor size is determined by the actual content of the image. Experimental results have demonstrated the feasibility of the MKD-SRC, but its performance is not satisfying. The rank-one recognition rate on FRGCv2.0+ database is only approximately 70% [34]. Later Zhang *et al*. [35] extended this method to 3D face recognition. They achieved high rank-one recognitino rate on different databases. However, multiple samples were still needed to form the gallery set.

Many studies on ear recognition have been conducted to date. However, as the results of the previous studies indicate, the OSPP problem remains unresolved. Most existing techniques require multiple training samples for recognition. SIFT-based matching algorithms have failed to achieve satisfying performance because unlike object recognition, ear recognition is an intra-class issue. ICP-based methods perform better but at the expense of high storage overhead and a high computational load involved in the recognition process. Thus, in this paper, we attempt to shed new light on the OSPP problem in ear recognition by fusing the 2D texture information and the 3D shape information into an SRC-based recognition framework.

## 2D and 3D Hybrid MKD-SRC

Inspired by the MKD-SRC face recognition approach [34], we propose a 2D and 3D hybrid method for ear recognition in this paper. The proposed method distinguishes itself from others as follows:

1. It is a robust alignment-free SRC based ear recognition approach that requires only OSPP in the gallery.
2. The hybrid MKD-SRC framework fuses 2D information with 3D information, and the 3D data are in the form of range images.

**Fig 1:**
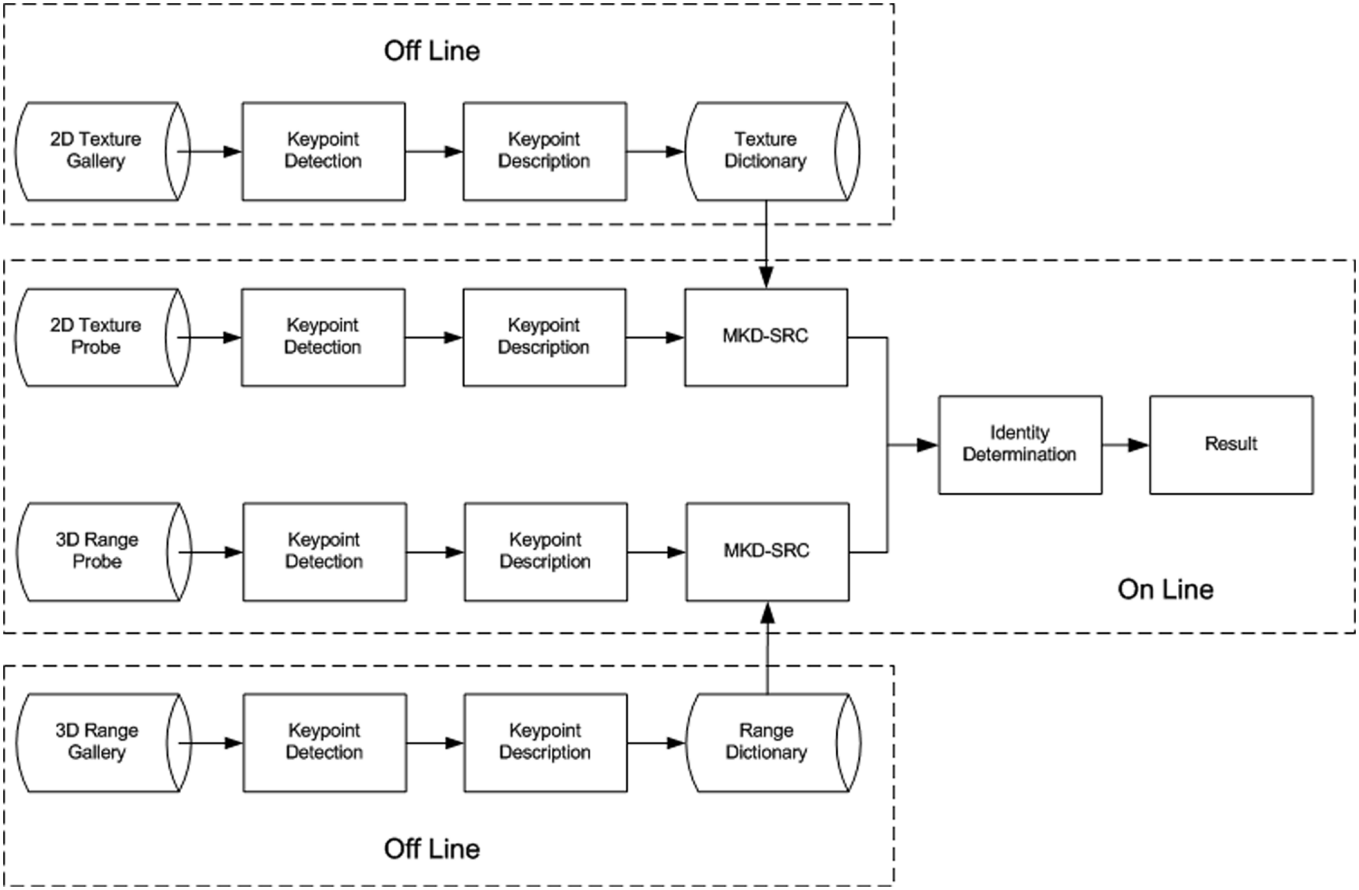
Overall flowchart of hybrid MKD-SRC.

The overall flowchart of our proposed hybrid MKD-SRC approach is shown in Fig 1.

### Database and Ear Region Extraction

**Fig 2:**
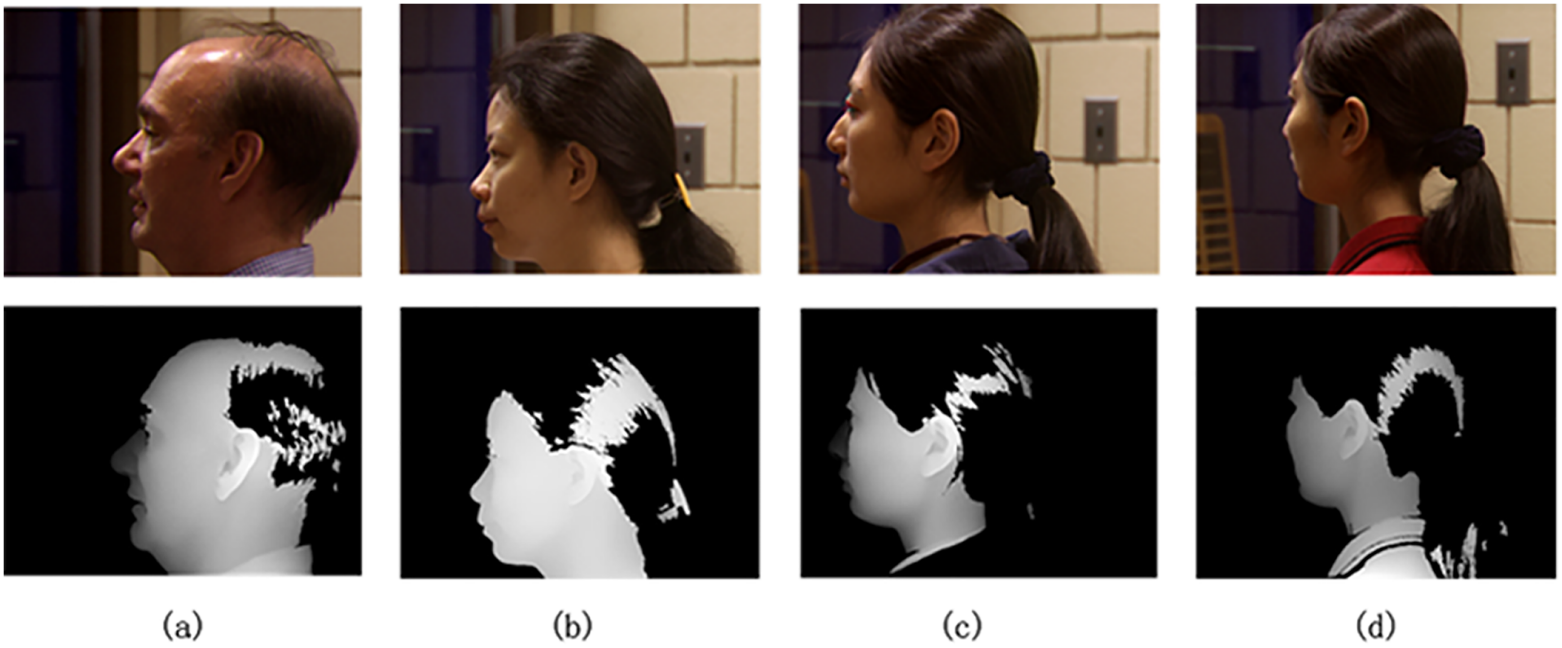
Examples of samples in UND collection J2. (a) A normal sample. (b) A sample with earrings. (c) & (d) Two samples of one person with a pose change.

The database used in our experiments is the University of Notre Dame (UND) collection J2 dataset [32], which includes 1,800 3D (+ corresponding 2D) profile images (containing ears) from 415 human subjects with an average 17.7-week time lapse. The 2D texture images and 3D pointcloud data were captured using a Minolta VIVID 910, and there are at least 2 samples for every subject. Fig 2 illustrates some examples of range images and corresponding texture images in the database, whose subject numbers are 02463, 04202 and 04221 respectively. All of the images were taken under rather loose conditions. Illumination variation, occlusion (including earrings and hair) and pose changes are common in this dataset, which are all significant challenges for any recognition techniques. In Fig 2c and 2d are of the same subject (No.04221), and there is a clear pose change between the two figures. Algorithms such as ICP would find it difficult to handle such a gallery-probe pair because the ear surfaces change considerably.

**Fig 3:**
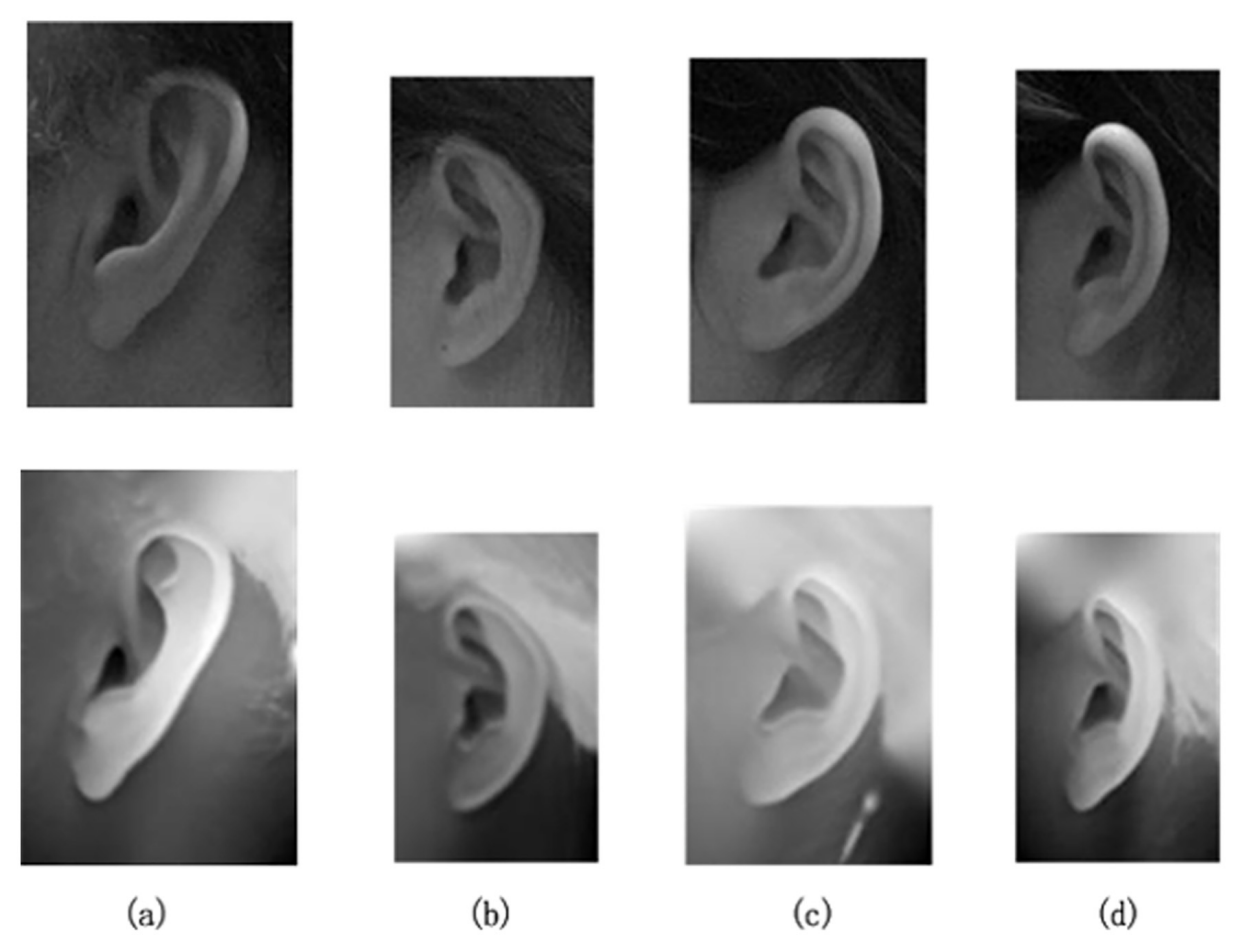
Examples of extracted ear regions.

Because ear region extraction is not the focus of this paper, we used the improved Adaboost algorithm [36] to extract the ear regions from the profile image of the 2D texture. Then, the corresponding 3D data of ear regions can be extracted at the same time. Fig 3 shows the results of the ear region extraction. The improved Adaboost algorithm is implemented on the 2D texture images alone, and then, the extracted 3D pointcloud data are represented in the form of range images. As seen from Fig 3, only two images per subject are needed in the gallery, i.e., a 2D texture image and 3D range image. These two images were taken at the same time and are only different representations of a single sample.

### Keypoint Detection and Description

Local features have recently gained increasing attention in the biometrics field because of their robustness to illumination and pose variations [37]. A local feature is an image pattern that differs from its immediate neighborhood [38]. With only OSPP available in the gallery, local feature is a useful tool to maximize the use of the only sample. The SIFT algorithm [39] is one of the most popular keypoint detectors and descriptors. It has been widely used in machine vision because of its robust repeatability property against image translation, rotation and scaling. However, SIFT is not invariant under affine transformation, and human ear images often suffer from pose changes (see Fig 3c and 3d for an example). Another limitation of SIFT is that it provides a small number of keypoints for ear images, which is not sufficient for identification because most ears look similar to each other.

**Fig 4:**
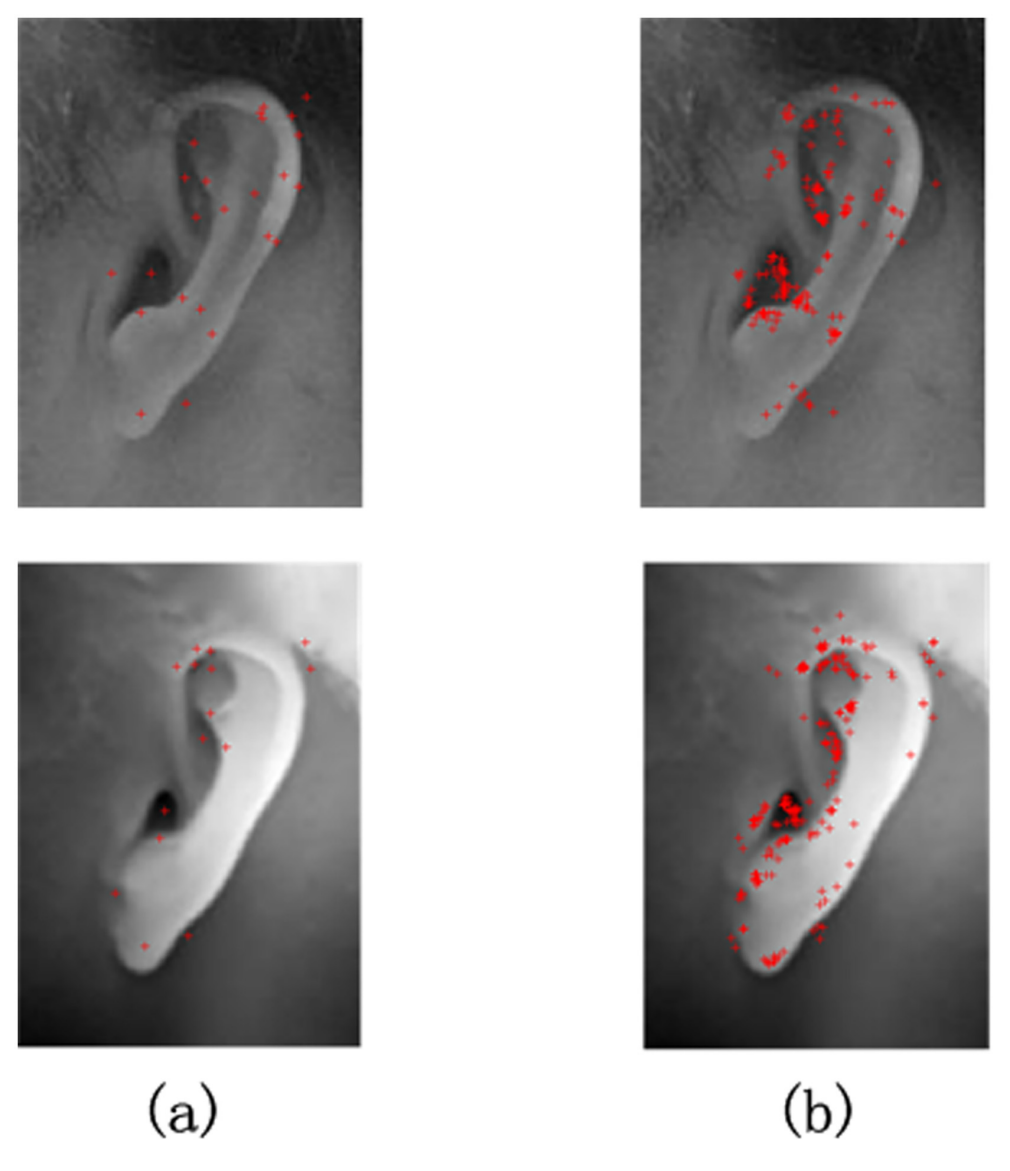
Comparison of SIFT and ASIFT on the same sample. (a) Result of the SIFT algorithm. (b) Result of the ASIFT algorithm.

Among all of the variants of SIFT, Affine-SIFT (ASIFT) [40] stands out for its fully affine invariance mathematically. SIFT is invariant to four out of the six parameters of an affine transform, whereas ASIFT is invariant to all the six by simulating all image views according to the two camera axis orientation parameters, namely, the latitudinal and longitudinal angles. Thus, ASIFT detects more keypoints than SIFT on the same image. For example, Fig 4 shows the results of keypoint detection given by SIFT and ASIFT. The keypoint numbers in Fig 4 are compared in Table 1.

**Table 1:** Comparison of numbers of keypoints from SIFT and ASIFT.

	2D image	3D image
**SIFT**	25	16
**ASIFT**	173	234

After each keypoint is described by a 128-dimensional descriptor, ASIFT matches the keypoints from a probe image to every sample in the gallery. In this case, 415 matches should be performed for each probe image, and keypoint mismatching is likely to occur during the process. Thus, we use only the 128-dimensional descriptor generated by the ASIFT algorithm instead without the matching step of the ASIFT algorithm. We believe that a robust classification technique can perform one-to-many recognition using only the ASIFT descriptors.

### Dictionary Construction

The dictionary is the core of an SRC-based recognition framework. We build two separate dictionaries, namely, a texture dictionary and a range dictionary, to use both 2D and 3D information. For one sample in the gallery, suppose that *k*_*Tc*_ keypoints from the 2D texture image and *k*_*Rc*_ keypoints from 3D range image are detected for subject *c* in the gallery. The corresponding *k*_*Tc*_ texture descriptors are denoted by **d**_*C*1_, **d**_*C*2_, …, **d**_*CkTc*_, and *k*_*Rc*_ range descriptors are denoted by **d**_*C*1_, **d**_*C*2_, …, **d**_*CkRc*_, where each descriptor is a 128-dimensional vector. Let

$$D_{Tc}=(d_{c1},d_{c2},\ldots ,d_{ckTc})$$ (1)

$$D_{Rc}=(d_{c1},d_{c2},\ldots ,d_{ckRc})$$ (2)

Then, a texture dictionary of all the *C* subjects is built as

$$D_{T}=(D_{T1},D_{T2},\ldots ,D_{TC})$$ (3)

In the same manner, a range dictionary of all the *C* subjects is built as

$$D_{R}=(D_{R1},D_{R2},\ldots ,D_{RC})$$ (4)

**Fig 5:**
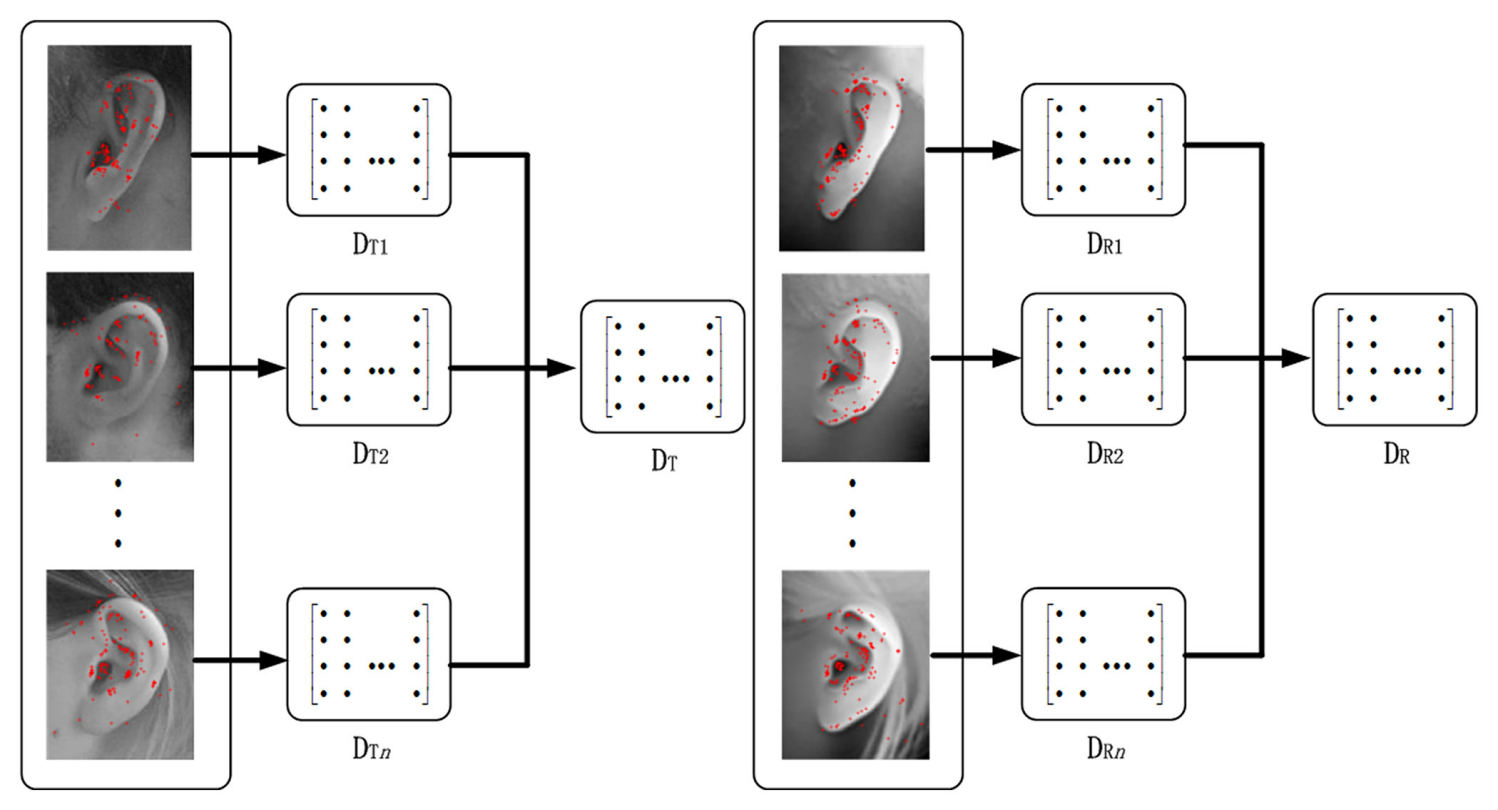
Illustration of the construction of hybrid MKD-SRC dictionaries.

Both **D**_*T*_ and **D**_*R*_ are quite large and are therefore over-complete. According to the theory of compressed sensing, a sparse solution is possible for an over-complete dictionary [41]. Therefore, any descriptor from the *C* subjects can be linearly represented in terms of **D**_*T*_ or **D**_*R*_. The construction of these two dictionaries is illustrated in Fig 5.

### Multitask Sparse Representation

Given a probe ear image (2D texture image or 3D range image) with *n* descriptors

$$Y=(y_{1},y_{2},\ldots ,y_{n})$$ (5)

the sparse representation problem is formulated as

$$\overset{\wedge }{X}=\text{arg }\underset{X}{\text{min}}\sum_{i=1}^{n}{\left\|x_{i}\right\|}_{0},\;s.t.Y=DX$$ (6)

Where **X** = (**x**_1_, **x**_2_,…,**x**_n_) ∊ *R* ^*K*×*n*^ is the sparse coefficient matrix, *K* is the total number of atoms in **D**, || ||_0_ denotes the $l_{0}$ norm of a vector, and **D** denotes either **D**_*T*_ or **D**_*R*_ corresponding to the type of probe image (similarly hereafter). The solution to this problem is Non-deterministic Polynomial-time hard (NP-hard). According to the theory of compressed sensing [42], sparse signals can be recovered with a high probability via $l_{1}$-minimization. Then, this problem can be solved by solving

$$\overset{\wedge }{X}=\text{arg }\underset{X}{\text{min}}\sum_{i=1}^{n}{\left\|x_{i}\right\|}_{1},\;s.t.Y=DX$$ (7)

Where ||·||_1_ denotes the $l_{1}$ norm. This is a multitask problem because **X** and **Y** have multiple columns. For each descriptor *y*_*i*_ in the probe image, we can solve the following set of *n* $l_{1}$ -minimization problems:

$${\overset{\wedge }{x}}_{i}=\text{arg }\underset{xi}{\text{min}}{\left\|x_{i}\right\|}_{1},\;s.t.y_{i}=Dx_{i},\;i=1,2,\ldots ,n$$ (8)

To solve Eq (8), a number of fast $l_{1}$ minimization algorithms have been proposed, including Homotopy [43], FISTA [44], DALM [45], SpaRSA [46], and $l_{1}\_l_{s}$ [47]. We use the Homotopy algorithm due to its relative balance between speed and accuracy. Because the *n $l_{1}$*-minimization problems in (8) are independent of each other, parallel computation can accelerate the algorithm.

Because the size (*K*) of the dictionary may be overly large, which makes solving (8) computationally expensive, we adopt the approximate solution proposed by Liao *et al* [34]. For each probe descriptor **y**_*i*_, we first compute the following linear correlation coefficients between **y**_*i*_ and all of the descriptors in the dictionary **D**:

$$c_{i}=D^{T}y_{i},\;i=1,2,\ldots ,n$$ (9)

Then, for each **y**_*i*_, we keep only *L* (*L*<<*K*) descriptors according to the top *L* largest values of *c*_*i*_, resulting in a smaller subdictionary $D_{128\times L}^{\left(i\right)}$. We take the suggestion of Liao *et al* [34] and set *L* = 100.

For further determination of the probe’s identification, we denote the reconstruction residual of subject *c* of the gallery as follows:

$$r_{c}(Y)=\frac{1}{n}{\sum_{i=1}^{n}\left\|y_{i}-D_{c}\delta_{c}(\overset{\wedge }{x_{i}})\right\|}_{2}^{2}$$ (10)

Where *δ*_*c*_(·) is a function that selects only the coefficients corresponding to subject *c*. *r*_c_(**Y**) measures the dissimilarity between the probe and subject *c* in the gallery by comparing the reconstructed vectors and probe descriptors.

### 2D and 3D Information Fusion and Identity Decision

As stated above, a probe sample (including a 2D texture image and its corresponding 3D range image) is entered into the algorithm, and we can obtain two reconstruction residual vectors, **r**_*T*_ and **r**_*R*_:

$$r_{T}={\left(r_{1}(Y_{T}),r_{2}(Y_{T}),\ldots ,r_{C}(Y_{T})\right)}^{T}$$ (11)

$$r_{R}={\left(r_{1}(Y_{R}),r_{2}(Y_{R}),\ldots ,r_{C}(Y_{R})\right)}^{T}$$ (12)

Sorting them in ascending order, we obtain the least and second least residual:${r^{\prime }}_{T}$, ${r^{\prime \prime }}_{T}$ and ${r^{\prime }}_{R}$,${r^{\prime \prime }}_{R}$. To fuse the 2D and 3D information, we can determine the probe’s identity as follows:

$$ID=\{\begin{array}{c} ID({r^{\prime }}_{T})\begin{array}{cc} & if\frac{{r^{\prime }}_{T}}{{r^{\prime \prime }}_{T}}<\frac{{r^{\prime }}_{R}}{{r^{\prime \prime }}_{R}} \end{array}\\ ID({r^{\prime }}_{R})\begin{array}{cc} & if\frac{{r^{\prime }}_{R}}{{r^{\prime \prime }}_{R}}<\frac{{r^{\prime }}_{T}}{{r^{\prime \prime }}_{T}} \end{array} \end{array}$$ (13)

Where $ID({r^{\prime }}_{T})$ denotes the identity based on 2D information and $ID({r^{\prime }}_{R})$ denotes the identity based on 3D information. By measuring the ratio of $r^{\prime }$ and $r^{\prime \prime }$, the confidence of the result given by either 2D information or 3D information is calculated. A lower $r^{\prime }/r^{\prime \prime }$ results in a higher confidence. The detailed analysis is presented in the following section. We refer to the overall algorithm as hybrid MKD-SRC.

## Experiment Results and Discussion

In this section, we evaluate the hybrid MKD-SRC algorithm on the UND J2 dataset to validate its feasibility and effectiveness. For each subject in the dataset, there are at least 2 samples of 2D texture images and 3D pointcloud data. We perform our experiment on all 415 subjects with one sample in the gallery and one sample for the probe. For those subjects that have more than two samples, we randomly select two from the available images. Then, we obtain a gallery set of 415 subjects and a test set of 415 subjects.

**Fig 6:**
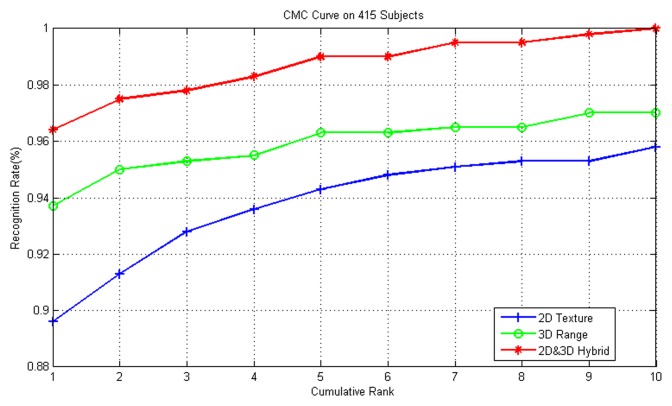
CMC curves on different data types.

First, we conduct our experiment on either the 2D texture images or 3D range images separately. Then, we combine both image types in the proposed hybrid MKD-SRC algorithm. The results are shown in Table 2. The cumulative match characteristic (CMC) curves of rank 10 from these three recognition experiments are presented in Fig 6.

**Table 2:** Rank-one reognition rates on different data types.

Data	Rank-1 Recognition Rate
**2D Texture**	89.6%
**3D Range**	93.7%
**2D&3D Hybrid**	96.4%

**Fig 7:**
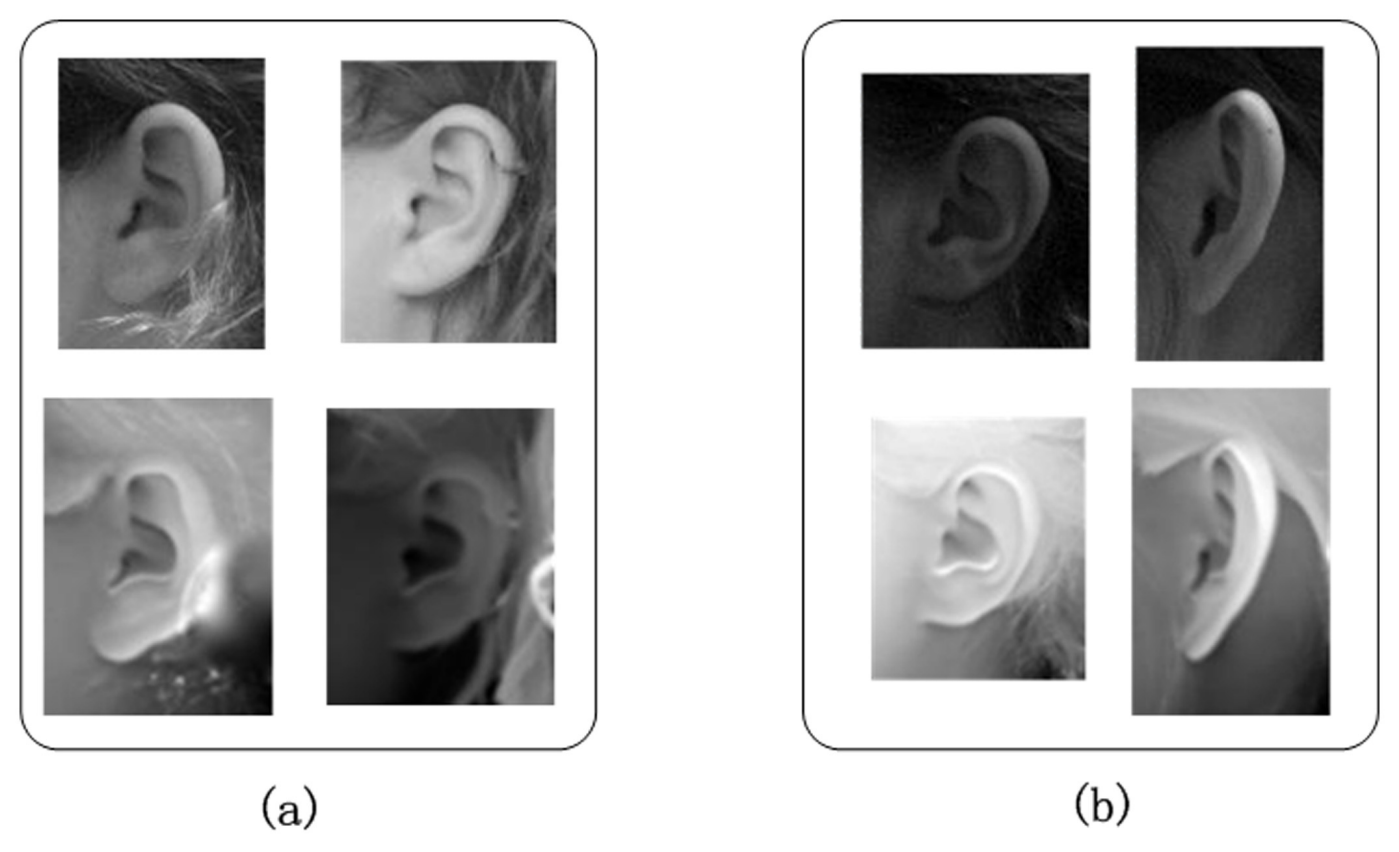
Examples of Case 1 and Case 2. (a) Case 1. (b) Case 2.

As is shown in Table 2 and Fig 6, the hybrid of 2D information and 3D information outperforms methods using either type of data separately. Additionally, among all of the samples in the test set, we look into the following 3 special cases:

Case 1. $ID({r^{\prime }}_{T})=ID_{real}$, but $ID({r^{\prime }}_{R})\neq ID_{real}$.
Case 2. $ID({r^{\prime }}_{R})=ID_{real}$, but $ID({r^{\prime }}_{T})\neq ID_{real}$.
Case 3. $ID({r^{\prime }}_{T})\neq ID({r^{\prime }}_{R})$, but *ID* = *ID*_*real*_.

where *ID*_*real*_ denotes the real identify of the sample and *ID* denotes the identity given by the hybrid MKD-SRC. In other words, Case 1 includes the samples recognized correctly by using 2D information but incorrectly by using 3D information. Case 2 includes the samples recognized correctly by using 3D information but incorrectly by using 2D information. Case 3 includes the samples for which different opinions are given by using 2D information or 3D information alone but are recognized correctly by the hybrid MKD-SRC. Some examples of Cases 1 and 2 are given in Fig 7.

As shown in Fig 7, Case 1 (Fig 7a) is primarily caused by the distraction of hair and earrings. The existence of these non-ear objects is misleading for the classification using range images whose gray value is the distance from the camera to the object. Thus, it is difficult for the algorithm to distinguish an earring or hair from the ear. However, the classification using 2D images can avoid this issue because of the relatively large difference between an ear and a non-ear object on the gray level. The experimental result reveals that 2D information is more robust against occlusion including hair and earrings for ear recognition. For Case 2 (Fig 7b), the main reasons for the failure of 2D information are illumination variance and pose changes. However, 3D information performs better under such conditions. To summarize, 2D texture images and 3D range images mutually complement each other in ear recognition. Using both types of images is useful for solving the OSPP problem, as demonstrated by the proposed hybrid MKD-SRC.

**Fig 8:**
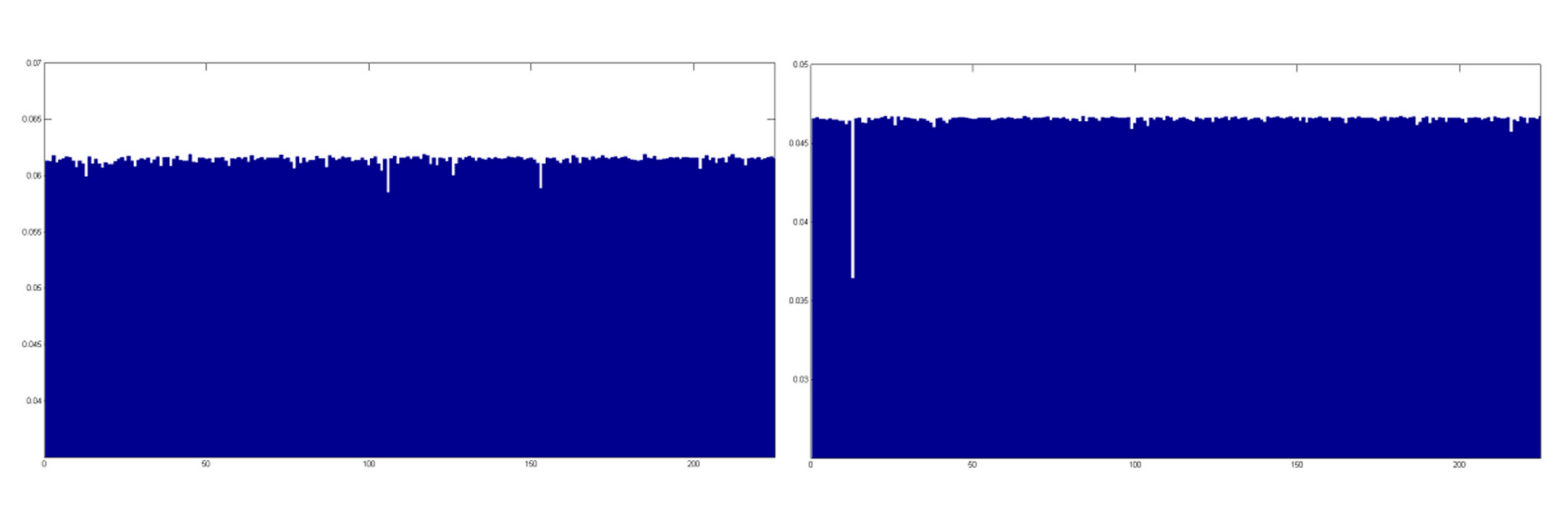
Example of reconstruction residuals of Case 3. (a) 2D texture image. (b) 3D range image.

When the findings from classification using 2D and 3D information disagree, which is the condition of Case 3, we adapt (13) to make the final decision. For example, Fig 8 illustrates reconstruction residuals of a probe sample (a) for the 2D texture image and (b) for the 3D range image. Only the region including the least and second least residuals are shown for clarity.

There is a larger difference between the least (${r^{\prime }}_{R}$) and second least (${r^{\prime \prime }}_{R}$) residuals in Fig 8b than that between the least (${r^{\prime }}_{T}$) and second least (${r^{\prime \prime }}_{T}$) residuals in Fig 8a, i.e., ${r^{\prime }}_{R}/{r^{\prime \prime }}_{R}<{r^{\prime }}_{T}/{r^{\prime \prime }}_{T}$. Because the reconstruction residual denotes the dissimilarity between the probe and object in the gallery, a smaller $r^{\prime }/r^{\prime \prime }$ indicates a higher confidence level. For the two findings that 2D and 3D information generate, we choose the one with a higher confidence. In this manner, the hybrid MKD-SRC algorithm fuses 2D and 3D information to complete a robust ear recognition framework against occlusion, illumination and pose changes with only one sample in the gallery.

In Table 3, we compare the proposed hybrid MKD-SRC with some typical and state-of-the-art published ear recognition studies. Zhang *et al*.’s SRC method [27] achieved a satisfactory recognition rate with a remarkably high speed. However, it required MSPP in the gallery, and the recognition rate decreased for fewer samples. For 5 samples per person in the gallery, the recognition rate was only 87.79% [27]. Although all ICP-based methods [31] [32] [33] required only OSPP in the gallery and offered high recognition rates, they required considerable time to match the probe to all samples in the gallery. One identification action needed hundreds of seconds. By contrast, the proposed hybrid MKD-SRC method shows superiority in the computational speed, and the recognition rate is competitive with the ICP-based methods.

**Table 3:** Performance comparison.

	Approach	Datasetsize	Data Type	Recognition Time	Number of SSGs^a^	Rank-1 RR^b^
**Mu *et al*. [9]**	LABSSFE	77	2D	-	1	85%
**Dewi & Yahagi [21]**	SIFT	-	2D	-	1	78.8%
**Kisku [20]**	Feature Level Fusion of SIFT	400	2D	-	1	94.31%
**Zhang *et al*. [26]**	SRC	85	3D	0.041s	10	95.23%
**Chen & Bhanu [29]**	LSP+ICP	302	2D&3D	1117s	1	96.7%
**Yan & Bowyer [32]**	ICP	415	2D&3D	2075s	1	97.6%
**Islam *et al*.[33]**	L3DF+ICP	415	2D&3D	946s	1	93.5%
**This paper**	Hybrid MKD-SRC	415	2D&3D	16.45s	1	96.4%

^a^SSG = Samples per Person in the Gallery.

^b^RR = Recognition Rate.

## Summary and Future Directions

We have addressed the OSPP problem of ear recognition and proposed a new SRC-based ear recognition approach fusing 2D and 3D information called the hybrid MKD-SRC, which is alignment-free and normalization-free. Our approach forms two dictionaries, namely, the texture dictionary and range dictionary, by detecting and describing keypoints of every sample in the gallery. A probe ear is sparsely represented by these two dictionaries, and a hybrid technique is adapted to fuse 2D and 3D information. In this manner, the identity of the probe ear can be inferred with only OSPP in the gallery. The proposed approach shows robustness to occlusion, illumination variation and pose changes. A rank-one recognition rate of 96.4% is achieved for the UND J2 database of 415 subjects, and the recognition speed is satisfactory compared to those of other methods.

We believe that one of the best ways to solve the OSPP problem is to maximize the use of local information of the only sample in the gallery. Thus, the representation of the samples is a key issue. In the future, we will explore potential ways to effectively represent the samples in the gallery.
